# Mechanisms of Mitochondrial Dysfunction in Lysosomal Storage Disorders: A Review

**DOI:** 10.3390/jcm9082596

**Published:** 2020-08-11

**Authors:** Karolina M. Stepien, Federico Roncaroli, Nadia Turton, Christian J. Hendriksz, Mark Roberts, Robert A. Heaton, Iain Hargreaves

**Affiliations:** 1 Adult Inherited Metabolic Diseases, Salford Royal NHS Foundation Trust, Salford M6 8HD, UK; 2 Division of Neuroscience and Experimental Psychology, School of Biology, Medicine and Health, University of Manchester and Manchester Centre for Clinical Neuroscience, Salford Royal NHS Foundation Trust, Salford M6 8HD, UK; federico.roncaroli@manchester.ac.uk; 3 School of Pharmacy, Liverpool John Moore University, Byrom Street, Liverpool L3 3AF, UK; nadia.turton@ljmu.ac.uk (N.T.); robert.heaton@ljmu.ac.uk (R.A.H.); I.P.Hargreaves@ljmu.ac.uk (I.H.); 4 Paediatrics and Child Health, Steve Biko Academic Unit, University of Pretoria, 0002 Pretoria, South Africa; chris@fymcamedical.co.uk; 5 Neurology Department, Salford Royal NHS Foundation Trust, Salford M6 8HD, UK; markrob@doctors.org.uk

**Keywords:** mitochondrial dysfunction, lysosomal storage diseases, oxidative stress, inflammation, reactive oxygen species, autophagy, mitophagy and cytokine

## Abstract

Mitochondrial dysfunction is emerging as an important contributory factor to the pathophysiology of lysosomal storage disorders (LSDs). The cause of mitochondrial dysfunction in LSDs appears to be multifactorial, although impaired mitophagy and oxidative stress appear to be common inhibitory mechanisms shared amongst these heterogeneous disorders. Once impaired, dysfunctional mitochondria may impact upon the function of the lysosome by the generation of reactive oxygen species as well as depriving the lysosome of ATP which is required by the V-ATPase proton pump to maintain the acidity of the lumen. Given the reported evidence of mitochondrial dysfunction in LSDs together with the important symbiotic relationship between these two organelles, therapeutic strategies targeting both lysosome and mitochondrial dysfunction may be an important consideration in the treatment of LSDs. In this review we examine the putative mechanisms that may be responsible for mitochondrial dysfunction in reported LSDs which will be supplemented with morphological and clinical information.

## 1. Introduction

Lysosomal storage diseases (LSDs) consist of a group of more than 70 inherited metabolic conditions caused by defects in genes that encode proteins involved in lysosomal homeostasis including lysosomal enzymes, non-enzymatic membrane proteins or non-lysosomal proteins [1]. The majority of LSDs are caused by impairment of the lysosomal acidic hydrolases, although, defective membrane trafficking and catabolite export together with impaired lysosome biogenesis can also cause a storage disorder [2]. The impairment of lysosomal function as a result of these aberrant proteins can lead to the accumulation of storage material(s) in endosomes and lysosomes, eventually compromising cellular functions [2]. The combined incidence of LSDs has been estimated to be approximately 1:5000 live births, although the true incidence is probably underestimated [1].

LSDs are disorders with autosomal recessive mode of inheritance while Fabry disease (FD), Danon disease, and Hunter syndrome have an X-linked inheritance pattern [3]. The age of onset and clinical symptoms vary for the different LSDs, although, neurological impairment is commonly associated with these disorders [4]. Together with LSDs, neurological dysfunction is also a common clinical manifestation of primary mitochondrial disease suggesting a connection between the function of these two organelles [5]. Lysosomes play a central role in a process known as autophagy. Autophagy is an intracellular mechanism by which macromolecules and organelles are transported to lysosome for degradation and recycling [6]. The autophagic degradation of mitochondria is known as mitophagy. This process enables the removal of dysfunctional mitochondria from the cell [5]. Mitophagy is important in post-mitotic cells such as neurons, which have limited capacity to up-regulate glycolytic ATP production. The removal of damaged mitochondria prevents the accumulation of reactive oxygen species (ROS) that are an inevitable consequence of impaired oxidative phosphorylation and may cause further impairment of mitochondria compromising cellular energy status [5]. Evidence of impaired mitophagy together with mitochondrial dysfunction has been reported in neural cells derived from a mouse model of Gaucher disease [7,8,9].

The acidic environment of the lysosomal lumen (pH from 5.2 to 6.1) is maintained predominantly by a V-ATPase. The enzyme uses the free energy liberated from the hydrolysis of ATP to pump protons into the lumen of the organelle [10]. Therefore, lysosomal acidification requires functioning mitochondria to provide the requisite ATP for this process, although no studies as far as the authors are aware have reported lysosomal dysfunction in patients with primary mitochondrial defects. However, a deficiency in the mitochondrial respiratory chain (MRC) electron carrier, coenzyme Q10 (CoQ10) has been reported in Mucopolysaccharidosis (MPS) patients [11]. In addition to impairing oxidative phosphorylation and depriving the V-ATPase of a source of ATP, a deficit in CoQ10 may also compromise the function of the lysosomal respiratory chain that is also required for maintaining the acidity of this organelle [10]. In the lysosomal respiratory chain CoQ10 functions as both an electron and proton carrier [10]. In view of the reported susceptibility of the lysosome to oxidative stress (OS) induced impairment, the antioxidant function of CoQ10 may also offer some protection to this organelle from free radical induced oxidation [10,11].

In view of the important relationship between the two organelles, therapeutic strategies that target mitochondrial dysfunction and/or aberrant mitophagy in LSDs may be important to consider in future treatment modalities.

The purpose of this review is to highlight evidence of mitochondrial dysfunction in LSDs and, highlight putative mechanisms and potential therapeutic strategies. In addition, case reports of patients with the LSDs, FD, and Pompe disease (PD) will be used to outline morphological and clinical evidence of mitochondrial dysfunction in these disorders.

## 2. LSDs Associated with Integral Lysosomal Membrane Proteins

### Niemann Pick C

Niemann Pick C (NPC) (OMIM #257220) is a slowly progressive neurodegenerative condition caused by faulty cholesterol trafficking resulting from mutations in *NPC1* or *NPC2* genes [12]. The *NPC1* or *NPC2* genes encode for the NPC1 membrane spanning protein and the NPC2 soluble lumen protein, respectively. The proteins are involved in cholesterol efflux from late endosomes and lysosome [12]. Mitochondrial dysfunction has been associated with NPC, although the actual cause of this impairment has yet to be fully elucidated and it may result from a number of different factors including oxidative stress and antioxidant capacity [13], increased mitochondrial cholesterol accumulation and a repression of mitochondrial biogenesis [14].

Evidence of oxidative stress (OS) in NPC has been indicated by increased levels of the oxidative stress marker, protein carbonyls in the livers of a mouse model of the disease (NPC mice) coupled with a decreased level of the cellular antioxidant, glutathione (GSH; [14]).

Increased levels of circulatory cholesterol oxidation products were also reported in NPC patients together with decreased plasma CoQ10 [15]. CoQ10 serves as an electron carrier in the MRC and also functions as an important lipid soluble antioxidant. A deficit of CoQ10 in NPC patients may therefore compromise their cellular antioxidant capacity [16]. A subsequent study Ribas et al. [17] detected elevated levels of lipid oxidation products (TBARS) and protein carbonyls in the plasma and fibroblasts from NPC patients.

Interestingly, elevated levels of α-tocopherol, the active antioxidant form of vitamin E were reported in the cerebellum and cortex of mouse models of NPC, although plasma levels of α-tocopherol in both mice and NPC patients were comparable to control levels [18]. It was suggested that this excess of cellular α-tocopherol might have a pro-oxidant effect and be a contributory factor to the OS associated with this disorder [14]. However, studies in NPC-affected fibroblasts and hepatocytes indicated that α-tocopherol appears to be sequestered in vesicles of lysosomal origin and therefore it is not available to contribute to the cellular antioxidant capacity [18]. A deficit in peroxisomal catalase activity (enzyme involved in the breakdown of the ROS, hydrogen peroxide into water and oxygen) was reported in the brain and liver of NPC mice together with a partial impairment of fatty acid β-oxidation capacity [19]. Peroxisomal impairment can precede clinical symptoms in mice suggesting that it may be a contributory factor to disease pathophysiology [19]. The defect in catalase activity may result from impairment in peroxisomal activation, although a deficiency in the activity of this enzyme has not been reported in NPC patients [17].

OS induced mitochondrial impairment as the result of oxidative stress exposure may be caused by oxidative damage to membrane phospholipids, enzymes and/or mitochondrial DNA [13]. Once impaired, MRC may also become a major site of ROS generation and contribute to oxidative stress induced cellular dysfunction [13].

In addition to oxidative stress, mitochondrial impairment in NPC has also been associated with an increase in the cholesterol content of the membranes of this organelle [20]. The study by Yu et al. [21] demonstrated a significantly increased level of cholesterol in the mitochondrial membranes of cortical neurons, astrocytes, and mitochondrial derived from the brains of NPC mice which was associated with decreased ATP synthase activity and cellular ATP levels. The relationship between mitochondrial cholesterol content and oxidative phosphorylation was further supported by the ability of the cholesterol chelator, methyl-cyclodextrin, to restore ATP synthesis in the NPC mouse brain mitochondria [21]. The increase in cholesterol content is thought to adversely alter the physical properties of the mitochondrial membrane reducing the proton motive force and membrane potential with a concomitant decrease in ATP synthetic capacity [21]. An altered mitochondrial membrane lipid composition was shown to be associated with impaired MRC function in Parkinson’s disease as the possible result of aberrant super-complex formation [22]. The accumulation of cholesterol in mitochondrial membranes was reported to impair the transport of GSH into mitochondria, as the transport of this tripeptide is carrier mediated and dependent on the inner membrane fluidity [23]. Indeed, decreased mitochondrial GSH status was reported in the brain and liver of NPC increasing the vulnerability of the MRC to OS induced dysfunction [24]. Cholesterol transport from the late endosome/lysosome to the mitochondrion appears to be independent of the NCP1 protein and mediated by the cholesterol-binding membrane protein, MLN64 whose expression was reported to be increased in NPC1 deficient cell lines [25]. Furthermore, an overexpression of MLN64 was found to increase mitochondrial cholesterol content and decrease the GSH content of the organelle as well as impairing its function [25].

Studies in fibroblasts derived from NPC patients and brain and liver tissue from NPC mice have shown that the transcription factors, KLF2 and ETV1 which repress the genes encoding mitochondrial proteins are up-regulated resulting in an inhibition of mitochondrial biogenesis [26]. Although the mechanism of action of KLF2 and ETV1 has yet to be fully elucidated it is thought to involve the down regulation of the transcription factor NRF1, which is a known as a positive regulator of mitochondrial biogenesis. It was suggested that the expression of KLF2 and ETV1 may be part of a signaling cascade in which lysosomal impairment represses the generation of organelles whose degradation requires the functioning of this organelle [26].

**Figure 1:**
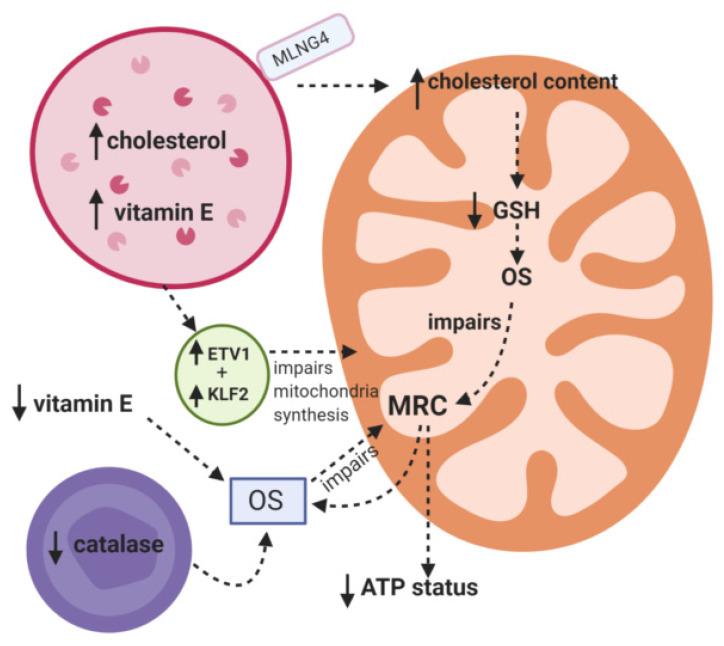
Putative mechanisms responsible for mitochondrial dysfunction in Niemann Pick C. MRC: Mitochondrial respiratory chain. ETV1: Transcription factor. KLF2: Transcription factor. OS: Oxidative stress. GSH: Reduced glutathione.

The putative mechanisms that have been implicated for MRC dysfunction in NPC are outlined in Figure 1.

## 3. LSDs Associated with Nonmembrane-Bound Lysosomal Hydrolases

### 3.1. Pompe Disease

Pompe disease (PD, glycogen storage disease type II) (OMIM #232300) is an autosomal recessive condition caused by mutations in the acid-α-glucosidase (GAA) gene. Deficiency of GAA enzyme leads to glycogen accumulation and an impairment of autophagy predominantly in skeletal and cardiac muscle [27].

Lysosomal dysfunction in PD results in incomplete autophagic flux and an accumulation of autophagic debris that is particularly prominent in muscle tissue [28,29]. In the absence of the disruptive autophagic build-up, recombinant enzyme GAA in enzyme replacement therapy (ERT) is able to clear lysosomal glycogen in muscle. The defective autophagy, however, affects trafficking and processing of the ERT [30,31,32,33]. Therefore the removal of this autophagic build-up enables efficient lysosomal glycogen clearance following ERT [34].

It is well documented that muscle pathology in untreated late-onset PD patients, can be heterogeneous ranging from the unaffected fibers to severe myopathic changes and considerable glycogen accumulation. The variability among PD patients can be explained by the differences in the levels of residual enzyme activity. In contrast, the unevenness of fiber involvement in each individual patient remains unexplained. Raben et al. [34] demonstrated that autophagic abnormalities are present in many muscle cells in late-onset patients (both juvenile and adults) [34].

Mitochondrial dysfunction was suggested to be a disease-modifying factor in PD. Fukuda et al. [30] reported that mitochondrial dysfunction is present in skeletal muscle pathology and that OS may also be associated with PD as indicated by an accumulation of lipofuscin, an autofluorescent material composed of oxidatively modified macromolecules detected in the lysosomes, autolysosomes (vesicles formed by autophagasomal–lysosomal fusion) and the cytosol of patients and animal models of the disease [29].

The source of the OS in PD is uncertain as yet. However, impaired mitophagy and disturbed calcium signaling may be contributory factors [29]. An impairment of the mitophagic process in PD is supported by the accumulation of damaged mitochondria in the cytosol of immortalized murine GAA knockout muscle cells (KO cells) [28,35]. The ROS generated by these defective organelles may then contribute to cellular OS as indicated in the study by Osellame et al. [7] which reported an association between defective mitophagy and cellular OS in a murine model of Gaucher disease.

An increase in cellular calcium concentration was reported in PD, which is thought to be the result of an up-regulation in L-type calcium channel expression. The mechanism inducing this increase in calcium channel expression is as yet uncertain, but it may be caused by the limited cellular glucose availability impairing the activity of the protein kinase, mammalian target of rapamycin complex 1(MTORC1) which can then result in an increase in calcium channel gene transcription. However, other mechanisms may be responsible for the increase in calcium channel expression [35]. The increase cytosolic calcium concentration in PD muscle cells saturates the mitochondria causing a decrease in its membrane potential as well as inducing a concomitant rise ROS generation [35]. Studies in murine KO muscle cells has indicated that the calcium overloading of the mitochondrion precedes lipofuscin accumulation, autophagic build-up and impaired MRC function [29].

Evidence of impaired MRC enzyme activities occurred in skeletal muscle tissue from two infants with PD. In this study undertaken by Selak et al. [36] the activities of MRC complex I, II, I-III, and II-III when expressed as a ratio to citrate synthase (CS) activity, the mitochondrial marker enzyme were found to be decreased compared to control levels. Interestingly, there was no evidence of aberrant mitochondrial morphology or proliferation to accompany the MRC enzyme deficiencies. Studies by Sarnat et al. [37] and Verity [38] have revealed evidence of excessive lipid accumulation in the skeletal muscle of PD patients indicating possible evidence of MRC dysfunction. However, no evidence of a deficiency in MRC complex II-III or IV activities was reported, although mitochondria were reported to have abnormal ultrastructural changes [38].

The etiology of cardiac complications associated with PD remains to be elucidated since it is difficult to obtain cardiac muscle compared with skeletal muscle. However, it has been suggested that OS may represent a major mechanistic driver of PD cardiomyopathy as indicated by a decreased GSH:GSSG ratio determined in late-onset PD induced pluripotent stem cell derived cardiomyocytes [39]. In addition, the antioxidant response element, NF-E2-related factor 2 (NRF-2) was also found to be down regulated in cardiomyocytes and skeletal muscle cells derived from a mouse model of PD [39].

Morphological studies indicated the presence of larger than normal mitochondria in skeletal muscle biopsy derived from a patient with adult onset PD was described by Engel and Dale [40]. These mitochondria, which were imperfect oval, polygonal, or prism shaped, contained dense granular material and paracrystalline inclusions located in the intercristae space [40,41,42]. Furthermore, functional mitochondria with swollen cristae have been also observed in induced pluripotent stem cells derived from the fibroblasts of two patients with PD [43].

Mitochondria were also found in autophagic vacuoles [36] in muscle biopsies of adult PD patients, these aberrant mitochondria remain sequestered in the vacuoles and are unable to reach lysosomes for recycling. It is still unclear whether mitochondrial abnormalities occur regardless or because of impaired autophagy, although calcium dysregulation and cellular OS may also be important contributory factor to consider [6].

**Figure 2:**
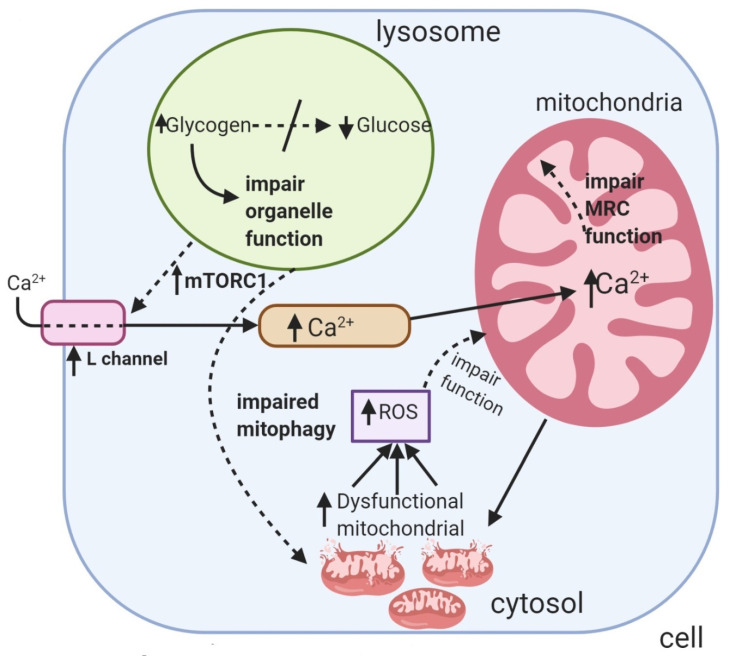
The putative mechanisms that have been implicated in inducing mitochondrial dysfunction in Pompe disease. MRC: Mitochondrial respiratory chain. Ca^2+^: Calcium. MTORC1: mammalian target of rapamycin complex 1. ROS: Reactive oxygen species.

**Figure 3:**
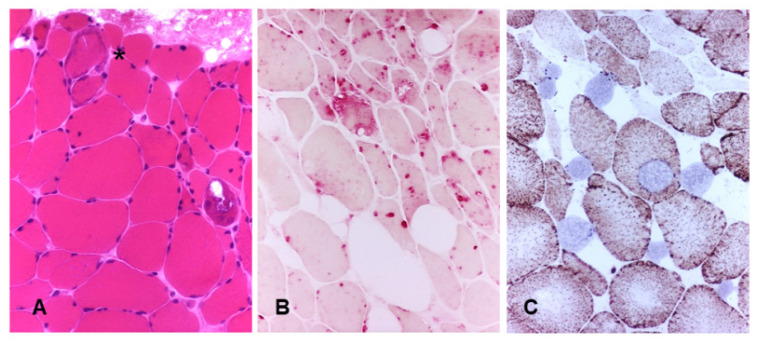
The muscle biopsy from a patient with late-onset Pompe disease (PD) shows myopathic changes including atrophic and hypertrophic fibers; one fiber with subsarcolemmal accumulation of mitochondria is also present (asterisk). (**A**)—hematoxylin-eosin, ×20; staining for acid phosphatase highlights the increase in lysosomes in muscle fibers. (**B**)—acid phosphatase, ×20; several COX-negative fibers (blue) are documented in the combined staining COX/SDH. (**C**)—COX/SDH, ×20.

The putative mechanisms that have been implicated for MRC dysfunction in PD are outlined in Figure 2 and mitochondrial changes in muscle biopsy from a PD patient is depicted on Figure 3. Mitochondrial changes in her muscles continued to be present despite ongoing ERT.

### 3.2. Mucopolysaccharidosis

Mucopolysaccharidosis (MPS; type I, II, III, IV, VI, VII) are disorders affecting the enzymes needed for the stepwise degradation of glycosaminoglycans (GAGs). The pathophysiology of MPS has not yet been fully elucidated, although, inflammation, oxidative stress (OS) and mitochondrial dysfunction are thought to be important contributory factors to the disease progression. A study utilizing a mouse model of MPS IIIC (OMIM #252930) provided insights into the disease mechanisms of MPS yielding evidence that may link neuroinflammation, mitochondrial dysfunction and neurodegeneration [44]. MPS IIIC is a severe neurologic disease caused by a genetic deficiency of heparan sulphate (HS) acetyl-CoA: a-glucosaminide *N*-acetyltransferase (HGSNAT) [44,45]. The result of the study indicated that HS and HS-derived oligosaccharide accumulating in the microglial cells may be the primary pathological event that triggers a cascade of reactions that eventually result in neuronal loss [44]. The HS and HS-derived oligosaccharides stored in the lysosomes of the microglia as well as the neurons to a lesser degree once released from the cells by exocytosis are then thought to induce a spectrum of inflammatory reactions in the brain by activation of the Toll-like receptors (TLR) present on the microglia [44,46]. The activation of the TLR receptors are then thought to induce the release of pro-inflammatory cytokines, MIP1a and TFN which were detected in brain tissue from the mouse model of MPS IIIC [44]. In addition, activated microglia also release ROS and reactive nitrogen species (RNS) including nitric oxide (NO) and peroxynitrite [47,48]. TFN induction of astrocytic inducible NO synthase activity may also be another source of RNS since activated astrocytes were detected throughout the brain of the mouse model of MPS IIIC [45,49]. The exposure of the neurons to the extracellular RNS and ROS may then have contributed to the progressive neuronal mitochondrial dysfunction reported in the animal model [44]. Evidence of neuronal mitochondrial impairment in this model was indicated by the presence of pleotropic and swollen mitochondria which were increased in number with many of them being swollen and containing disorganized cristae [44]. A study by Doll et al. [50] utilizing immortalized mouse hippocampal cells has indicated that MRC impairment may be caused by direct exposure to TFN irrespective of the mitochondrial toxicity induced by ROS and RNS. Although, its mechanism of action is uncertain, the loss of mitochondrial membrane potential associated with TFN treatment indicates the ability of this cytokine to induce a disruption of the structural integrity of the mitochondrial membranes [50]. Commensurate with its potential mitochondrial toxicity, TFN treatment is also associated with an increase in mitochondrial ROS generation [51]. The exposure of neurons to both external and internal sources of OS may explain the progressive mitochondrial dysfunction reported in the mouse model of MPS IIIC, which was also accompanied by a reduction in neuronal density [44]. In addition to morphological abnormalities, potential mitochondrial dysfunction in MPS IIIC has been indicated by the decreased MRC complex II and IV activities detected in brain mitochondria isolated from the MPS IIIC mouse model at the latter stages of the disease which was accompanied by a deficit in cerebral CoQ10 status [44,45].

Interestingly, a deficit in plasma CoQ10 status was also reported in MPS patients, including those with MPS IIIC [11]. It was proposed that the reduction in circulatory CoQ10 status may be related to the low level of vitamin B6 detected in the patients. The active form of vitamin B6, pyridoxal 5-phosphate, is required for the transamination of tyrosine into 4-hydroxyphenylpyruvic acid, an essential step in CoQ10 biosynthesis [11]. GAGs which are known to accumulate in MPS disorders may bind vitamin B6 reducing its circulatory levels [11]. The cause of the cerebral CoQ10 deficiency reported in the MPS IIIC mouse is as yet uncertain; however, it may be associated with the evidence of OS reported in MPS patients and animal model of the disease [52]. A study by Trudel et al. [53] using a mouse model of MPS IIIB (OMIN #252920) suggested that cerebral OS associated with this disorder may not be a secondary consequence of neuroinflammation but rather as a direct result of cellular HS accumulation, although, the mechanism(s) has yet to be elucidated.

An impairment of neuronal autophagosome–lysosome fusion and consequently, mitophagy was indicated in the mouse model of MPS IIIC which would account for the progressive accumulation of gangliosides, subunit c of mitochondrial ATP synthase (SCMAS) aggregates and deformed and dysfunctional mitochondria which have been detected in the advanced stages of the disease [44]. It was suggested that GAG accumulation impairs the lysosomal functions required to support autophagic degradation, although the mechanism(s) has yet to be fully elucidated [54]. The accumulation of dysfunctional mitochondria as a consequence of impaired mitophagy would also be expected to be a source of ROS generation contributing to neuronal OS [55].

Another MPS disorder, Maroteaux–Lamy syndrome (MPS VI), is caused by a deficiency of the lysosomal enzyme *N*-acetylgalactosamine-4-sulfatase [54]. It has been demonstrated that excessive dermatan sulphate accumulation alters the lysosomal ability to degrade cytoplasmic components or organelles through autophagy. Lysosomal storage leads to reduced functionality of lysosomes and consequently, autophagy deregulation [56]. Tessitore et al. [54] have shown evidence of impaired autophagy with increased levels of autophagic proteins, increased polyubiquitination and an accumulation of aberrant mitochondria within the autophagic vacuoles in human MPS VI fibroblasts as well as in affected tissues of an MPS VI rodent model. In vivo, this was associated with inflammation and apoptosis. Abnormal autophagy was described in other LSDs, proving that common mechanisms are downstream of different genetic defects in LSDs, including Mucolipidosis type II and IV (Table 1). The impairment in autophagy resulting in an accumulation of aberrant mitochondria would be expected to cause an increase in cellular ROS generation and concomitant cellular dysfunction as has been suggested for MPS III C [54].

**Figure 4:**
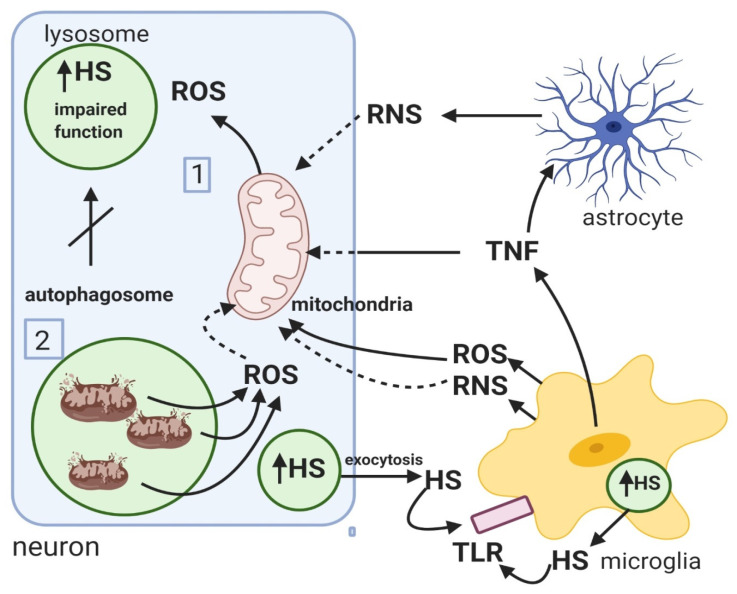
Putative mechanisms responsible for mitochondrial dysfunction in Mucopolysaccharidosis. HS: Heparin sulphate. MRC: Mitochondrial respiratory chain. ROS: Reactive oxygen species. RNS: Reactive nitrogen species. TNF: Cytokine.

The putative mechanisms that have been implicated for MRC dysfunction in MPS according to studies in patients and animal and cell models of the disease are outlined in Figure 4.

### 3.3. Fabry Disease

Fabry Disease (FD) (OMIM #300644) is an X-linked lysosomal storage disorder caused by the deficiency of the enzyme alpha-galactosidase A. It results in progressive intracellular deposition of globotriaosylceramide (GB3) and related neutral glycosphingolipids in multiple organ systems, including skin, kidneys, vascular endothelium, ganglion cells of the peripheral nervous system, and heart [88].

Several studies suggested that OS may be implicated in the pathophysiology of FD, particularly the cardiovascular involvement of the disease. GB3 accumulation was reported to cause a dose dependent increase in ROS production in cultured vascular endothelial cells from FD patients [89]. Interestingly, culturing endothelial cells in plasma from FD patients significantly increased ROS generation when compared with plasma from non-FD controls, although this effect was not found to be dependent upon GB3 levels suggesting that other factors in the plasma may also be responsible for inducing OS [89]. A subsequent study by Biancini et al. [90] reported evidence of OS and inflammation in the plasma of FD patients receiving ERT as indicated by markers of lipid and protein oxidation together with the pro-inflammatory cytokines, IL-6 and TNF. Furthermore, erythrocyte GSH status was found to be decreased in the FD patients together with a reduction in glutathione peroxidase activity and an elevated superoxide dismutase (SOD): catalase activity ratio [90]. The authors speculated that this altered SOD: catalase activity ratio may lead to an increased availability of the ROS, hydrogen peroxide. Urinary GB3 was found to be correlated with the plasma markers of both OS and inflammation which indicates an association between GB3 and the pro-oxidant and pro-inflammatory states of FD [90]. An accumulation of circulatory levels of GB3 and its deacetylated form, lyso-GB3 are thought to induce inflammation by binding to and activating the TLR receptors of the dendritic cells and as a consequence cause the generation of ROS and RNS [91]. A study by Tseng et al. [92] reported that the protein level of the mitochondrial antioxidant enzyme, SOD-2 was down regulated in FD-specific human induced pluripotent stem cells which was found to be associated with an increase in ROS generation together with an enhanced cellular accumulation of GB3.

GB3 treatment of human microvascular cardiac endothelial cells was reported to decrease the expression of NO synthase (eNOS), whereas, the expression of inducible NO synthase (iNOS) was found to be increased [93]. Moreover, alpha-galactosidase A deficiency has been associated with increased nitrotyrosine (a marker of reactive nitrogen species, RNS production) expression in aortic endothelial cells isolated from a mouse model of the disease [94]. Increased nitrotyrosine levels were also detected in skin biopsies and brain tissue from FD patients [95,96]. More recently, Shu et al. [97] demonstrate that alpha-galactosidase A knockdown by RNA interference in a human endothelial cell line results in GB3 accumulation, reduced endothelial NO synthase activity, and dramatically enhanced nitrotyrosine production. In addition, elevated levels of nitrotyrosine were detected in the plasma and aortic tissue of a alpha-galactosidase A knockout mouse, as well as in the plasma of untreated male patients with FD [97]. GB3 was reported to be widely distributed in the cell and be present in other organelles such as the nucleus and endoplasmic reticulum rather than being confined to the lysosome [98]. The presence of GB3 in the nucleus may be an explanation for its ability to induce iNOS whilst inhibiting the expression of eNOS [93]. iNOS produces large amounts of NO in comparison to eNOS, which is highly reactive with other free radicals. NO reacts with superoxide to form peroxynitrite, and this RNS can induce oxidative damage to biomolecules as well as the nitration of proteins explaining the high level of nitrotyrosine detected in FD patients as well as animal and cell models of the disease [99]. The loss of eNOS activity in FD disease which is essential for the maintenance of vascular homeostasis may contribute to the risk of incipient vasculopathy and provide a pathologic milieu for the accelerated development of cardiovascular complications associated with this disorder [93]. Therefore, nitrotyrosine represents a potential biomarker for vasculopathy in FD as well as indicating evidence of RNS generation [97]. GB3 induced ROS and RNS production was reported to contribute to cardiac dysfunction as well as promote cardiomyocyte protein nitration and DNA damage in FD [96].

In addition to oxidative and nitrosative stress as well as inflammation, evidence of MRC dysfunction was reported in FD. Lücke et al. [100] reported decreased MRC complex II, IV, and V activities in fibroblasts from FD patients compared to controls. The molecular mechanism responsible for the loss of MRC enzyme activity was not investigated in the study by Lücke et al. [100] and the accumulation of lysosomal storage (GB3) material may have caused the mitochondrial dysfunction. However, the possibility arises that the elevated levels of ROS and RNS associated with FD may have also contributed to the MRC impairment in view of their ability to cause oxidative damage to mitochondrial proteins, lipid and DNA [42]. In vivo studies were also documented impaired oxidative phosphorylation as indicated by the decreased level of the high energy phosphate molecules, ATP and creatine phosphate detected in the heart of FD patients [101]. Subsequent ERT was reported to result in a partial restoration in the cardiac level of these molecules.

Variation in mitochondrial DNA (mtDNA) haploid groups amongst FD patients may account for the differences in the susceptibility of individuals to ROS induced mitochondrial dysfunction as well as the ability of MRC to generate sufficient ATP via oxidative phosphorylation during cellular morbidity [102]. The susceptibility of mitochondria and mtDNA to OS induced dysfunction OS may also vary between tissues, thus modulating the phenotype and the natural course of the disease. Although certain haploid groups were shown to be more prevalent in patients, there was no observed correlation with gender, age of onset, or organ involvement [103].

**Figure 5:**
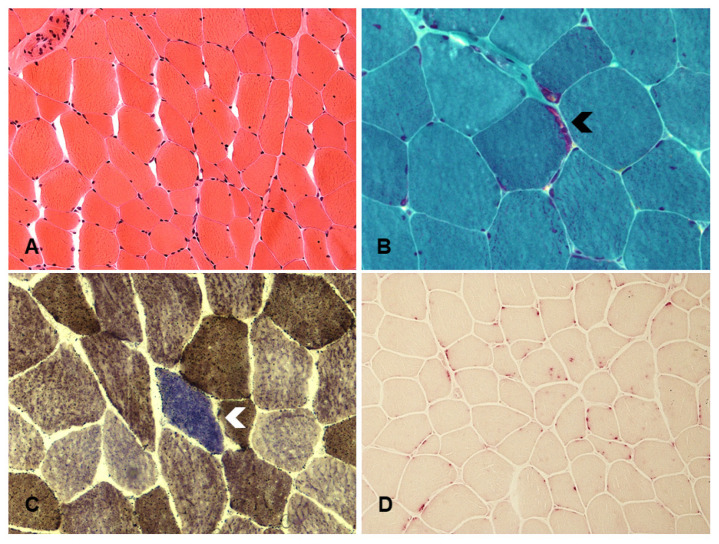
These images document the muscle from a patient with Fabry’s disease on ERT treatment; the biopsy is characterized by abnormal variation in fibre size and a few fibres showing internal nucleation (**A**, hematoxylin-eosin, ×20); focal subsarcolemmal accumulation of mitochondria but no ragged red fibres is seen (arrow) (**B**—modified Gomori’s trichrome, ×40); a COX-negative fibre is present (blue fibre, arrow) (**C**—COX/SDH staining, ×40); there is increase staining with acid phosphatase (red precipitate) that due to subsarcolemmal deposits of lipofuscins and increase in lysosomes (**D**—acid phosphatase, ×20).

The impaired mitochondrial energy supply in skin fibroblasts from a patient with FD is depicted on Figure 5. Compromised mitochondrial function may play a role in the pathogenesis of FD and is present despite of ERT (Figure 5). Fatigue and myopathy may be attributed to mitochondrial changes rather than FD itself. A trial of CoQ10 has been recommended with a variable outcome (KMS: personal observation).

**Figure 6:**
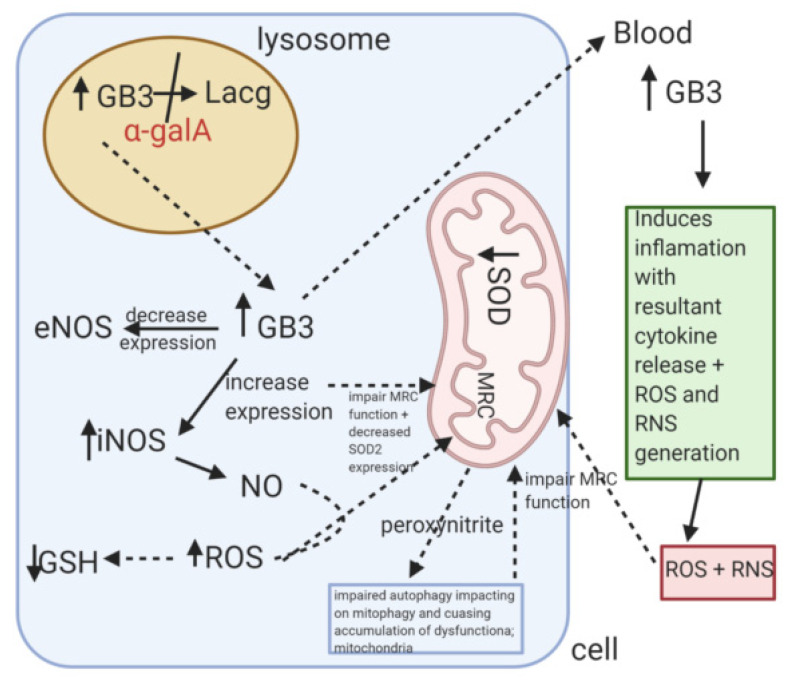
Putative causes of mitochondrial dysfunction in Fabry disease. GB3: globotriaosylceramide. Lacg: Lacoglyceramide. α-galA: alpha-galactosidase A. SOD-2: Superoxide dismutase-2. eNOS:endothelial nitric oxide synthase. iNOS: inducible nitric oxide synthase. MRC: Mitochondrial respiratory chain. GSH: Reduced glutathione. ROS: Reactive oxygen species. RNS: Reactive oxygen species. NO: Nitric oxide.

The putative mechanisms that have been implicated for OS and MRC dysfunction in FD are outlined in Figure 6.

## 4. Discussion

Mitochondrial dysfunction is emerging as an important contributory factor in the pathophysiology of LSDs. It is unsurprising given the symbiotic relationship between the two organelles although the molecular mechanisms governing this co-operation have yet to be fully elucidated. The impairment of lysosomal function in LSDs can directly impact upon the mitochondrion by perturbation of mitophagy [7]. The accumulation of mitochondria with impaired MRC function, as has been reported in LSDs, can cause an increased generation of cellular ROS and result in oxidative damage to the organelles including the lysosome as well as further impair mitochondrial function since the MRC is very vulnerable to OS induced inactivation. In addition to OS, which has been widely reported in LSDs and from sources other than the mitochondria, MRC dysfunction may also be caused from inhibition by macromolecules and protein aggregates which accumulate in the cytosol as a consequence of impaired autophagy [7]. Furthermore, MRC dysfunction in LSDs may also result from the alteration in lipids of the inner mitochondrial membrane (e.g., in NPC1) [21] or as the result of defects in calcium homeostasis (e.g., in PD) [104]. MRC dysfunction in LSDs, will eventually deprive the cell of ATP, which apart from being essential for maintaining lysosomal acidity will also cause cellular morbidity. Therefore, given the profound mitochondrial dysfunction that has been reported in some LSDs [68], treatment strategies that target the mitochondria may prove to be of therapeutic benefit to patients. Therefore, in view of the susceptibility of the MRC to OS induced impairment, a number of studies on antioxidant treatment have been undertaken employing both patient cells and animal models of LSDs. It is unknown however whether a similar response would be observed in clinical trials. It is known that even for primary mitochondrial diseases in which a CoQ10 deficiency is demonstrated in vitro, oral supplementation does not yield attainable clinical response for those patients.

A study utilizing Gaucher disease patient fibroblasts reported that combined pharmacological chaperone therapy with CoQ10 treatment was able to increase glucocerebrosidase activity and improve mitochondrial function [105]. Currently, there is a clinical trial to assess the therapeutic efficacy of *N*-acetyl cysteine (trial NCT03759639), the GSH precursor in NPC which is running at the National Institute of Health Clinical Centre in USA [106], although there is no indication when the results will be available.

Matalonga et al. [107] reported that treatment with both CoQ10 and antioxidant cocktail (α-tocopherol, *N*-acetylcysteine and α-lipoic acid) was able to significantly decrease GAG accumulation in selected MPS III patient fibroblasts. Apart from CoQ10, other products were investigated in MPS disorders. Genistein, a natural isoflavone, was considered an autophagy stimulator in experimental studies [108]. Trehalose, another autophagy stimulator, was shown to improve symptoms in MPS IIIB mice [109]. It was, therefore, postulated that autophagy can be profitable at least for animals affected with MPS as shown in vitro studies [110]. Strategies that enhance autophagy may provide judicious in the treatment of LSD as well as utilizing treatments that maximize residual MRC activities [10,79]. The enhancement of TFEB (master gene for that regulates lysosomal biogenesis) expression may prove beneficial to LSD patients since this has the potential to enhance mitophagy by maintenance of autophagy-lysosome pathway in addition to its ability to also induce mitochondrial biogenesis [79,110]. However, since multiple cellular pathways are impaired in LSDs, therapeutic strategies to ameliorate OS, improve mitochondrial function, and enhance autophagy combined with ERT may prove beneficial in the treatment of these disorders. Some other therapeutic approaches that target OS or mitigate mitochondrial dysfunction are available. One of these compounds is the CoQ10 analogue, idebenone, which as well as functioning as an antioxidant is able to mediate electron flow from the cytosol to complex III of the MRC and therefore bypass an MRC complex I deficiency [111]. Another compound to consider is the synthetic quinone, EPI-743 which has been reported to replenish GSH levels in both cell and human studies [111]. Thiamine (vitamin B1) may also be appropriate to increase the activity of pyruvate dehydrogenase, and thus enhance the oxidase decomposition of pyruvate [86]. Elamipretide is an aromatic cationic tetrapeptide, which has been reported to selectively bind to cardiolipin, a phospholipid component of the inner mitochondrial membrane which is required for optimal MRC activity and is able to protect it from oxidative damage [86]. Elamipretide inhibits the opening of mitochondrial permeability transition pore and enhances MRC function and is now used as a drug for the treatment of primary mitochondrial myopathy [86]. However, the use of appropriate markers to indicate evidence of mitochondrial function and OS as well as to monitor therapeutic intervention in LSDs will also prove to be of similar importance.

## Figures and Tables

**Table 1:** Mitochondrial dysfunction in lysosomal storage diseases.

Disorder	Enzyme Deficient/Protein Affected	Genetic Defect	Storage Material/Pathologic Hallmark	Domain of Mitochondrial Dysfunction	References
**Lysosomal Storage Disorders (LSDs) Associated with Integral Lysosomal Membrane Proteins**					
**Niemann Pick C**	Glycosylated transmembrane protein of the lysosomal membrane	*NPC1* and *NPC2* gene	Intracellular accumulation of cholesterol and other lipids like glycosphingolipids, sphingomyelin and sphingosine in the lysosomes and late endosomes	Autophagosomes accumulation and autophagy dysfunction in disease pathogenesis.	[57,58,59]
				Induction of autophagy.	
				Lysosomal cholesterol and lipid export, foam cells in visceral organs and neuronal storage.	
**Mucolipidosis IV**	TRPML-I (ion channel- mucolipin 1)	*MCOLN1* gene	Lipofuscin in lysosomes, lipids	Autophagosomes accumulation and authophagy dysfunction in disease pathogenesis.	[57,60,61,62,63]
				Block of authophagosome-lysosome maturation.	
**Cystinosis**	Reduced or absent function of the specific carrier cystinosin	*CTNS* gene	Cystine in lysosomes	Mitochondrial fusion is mediated by three key regulatory fusion proteins: the dynamin-related GTPases mitofusin 1 (MFN1) and mitofusin 2 (MFN2), and the dynamin-related GTPases optic atrophy 1 (OPA1); altered endosomal trafficking, impaired autophagy, and cell oxidation	[64,65]
**Danon disease**	LAMP-2 lysosomal membrane protein	*LAMP-2* gene	Glycogen	Autophagic flux, which results in excessive oxidative stress, and subsequent cardiomyocyte apoptosis; autophagosome accumulation, autophagy dysfunction in disease pathogenesis, block of autophagosome–lysosome maturation.	[57,66,67]
**LSDs Associated with Nonmembrane-Bound Lysosomal Hydrolases**					
**Pompe**	alpha-Glucosidase	*GAA* gene	Glycogen	Autophagosomes accumulation and autophagy dysfunction in disease pathogenesis;	[30,57,59]
				(Autophagic) accumulation in type II muscle fibers	
**Mucopolysaccharidosis: III A**	heparan *N*-sulfatase	*SGSH* gene	Heparan sulphate	Autophagosomes accumulation,	[44,56,57,68,69]
	*N*-acetyl-alpha-D-glucosaminidase				
**IIIB**	acetyl-CoA: aglucosaminide acetyltransferase	*NAGLU gene*		autophagy dysfunction in disease pathogenesis,	
				block of autophagosome–lysosome maturation;	
**IIIC**	*N*-acetylglucosamine-6-sulfate sulfatase	*HGSNAT gene*		Accumulation, Fragmentation/swelling, Selective reduction of OXPHOS complexes,	
**IIID**		*GNS gene*		Decreased coenzyme Q10	
**Mucopolysaccharidosis VI (Maroteaux–Lamy)**	*N*-acetylgalactosamine-4-sulfatase	*ARSB* gene	Dermatan sulphate	Autophagosome–lysosome fusion is not completely blocked; accumulation of dysfunctional mitochondria; increased levels of autophagic proteins, increased polyubiquitination and abnormal mitochondrial function	[54]
**Multiple Sulphatase Deficiency**	Sulphate esters (At least 7 lysosomal sulfatases and a microsomal sulfatase)	*SUMF1* gene	-	Autophagosomes accumulation, autophagy dysfunction in disease pathogenesis, induction of autophagy. Accumulation, Fragmentation, Decreased ATP content	[56,57,68,70]
**Mucolipidoses II**	*N* acetylglucosaminylphosphotransferase resulting in multiple enzyme deficiencies	*GNPTAB* gene	Mucopolysaccharides, lipids, glycoproteins	Autophagic and mitochondrial impairment.	[71,72,73]
**Mucolipidosis III**				Dysfunctional autophagosome, vacuolization (accumulation of autolysosomes)	[74]
**Gangliosidosis:** **GM1**	beta-galactosidase	*GLB1* gene	Gangiosides	Autophagosomes accumulation, autophagy dysfunction is disease pathogenesis, induction of autophagy.	[57,75,76]
**GM2**	beta-hexosaminidase	*HEXB gene*			
**Fabry disease**	alpha-galactosidase A	*GLA* gene	globotriasylceramide (Gb3)	Increased vacuoles level, reduction of mitochondrial activity, Lipid storage in endothelial and smooth muscle cells of blood vessels	[59,77]
**Farber disease**	Ceramidase	*ASAH1* gene	Ceramides, mucopolysaccharides, gangliosides	Ceremide/cholesterol structures accumulate in late endosomes and lysosomes, in mitochondria and the plasma membrane	[78]
**Gaucher disease**	glucocerebrosidase (*GBA1*)	*GBA1* gene	Glycosphingolipids, Glucosylceramide, GM1, GM2, GM3, GD3, Glucosylsphingosine	Impaired autophagy and proteasomal degradation pathways and mitochondrial dysfunction; pathological [Ca^2+^]c responses and delayed calcium deregulation, collapse of mitochondrial membrane potential and an irreversible fall in the ATP/ADP ratio; lipid storing macrophages	[7,8,79,80,81,82]
**Krabbe disease**	Galactosylceramidase	*GALC* gene	galactosyl-ceramide and galactosyl-sphingosine (psychosine)	Mitochondrial morphology ad accumulation,	[68,83,84]
				calcium signaling, oxidative stress and macromolecules accumulation in mitochondria;	
				Elevated ROS/reduced GSH,	
				Ca^2+^ overload,	
				Cytochrome c release	
**LSDs Associated with Other Defects**					
**Neuronal Ceroid Lipofuscinosis (Batten’s disease)**	Lysosome protease deficiencies	*CLN1–CLN12* gene	Lipofuscin	Active regulation of ATP synthase by Ca^2+^ is maintained in fibroblasts from CLN 2 and CLN3 but absent in those from CLN1. Autophagosome accumulation, autophagy dysfunction in disease pathogenesis, induction of authophagy.	[57,68,85,86,87]

## Abbreviations

CoQ10	Co-enzyme Q10
ERT	enzyme replacement therapy
LSD	lysosomal storage diseases
MRC	mitochondrial respiratory chain
NO	Nitric oxide
iNOS	inducible nitric oxide synthase
NPC	Niemann Pick C
OS	oxidative stress
ROS	redox oxidative stress
SCMAS	Subunit c of mitochondrial ATP synthase
SOD	Superoxide dismutase
V-ATPase	Lysosomal ATPase
TLR	Toll-like receptor
